# The life-extending effect of dietary restriction requires Foxo3 in mice

**DOI:** 10.1111/acel.12340

**Published:** 2015-03-23

**Authors:** Isao Shimokawa, Toshimitsu Komatsu, Nobutaka Hayashi, Sang-Eun Kim, Takuya Kawata, Seongjoon Park, Hiroko Hayashi, Haruyoshi Yamaza, Takuya Chiba, Ryoichi Mori

**Affiliations:** Department of Pathology, Nagasaki University School of Medicine and Graduate School of Biomedical SciencesNagasaki, Japan

**Keywords:** cancer, dietary restriction, Foxo, lifespan, longevity, mouse

## Abstract

Forkhead box O (Foxo) transcription factors may be involved in the salutary effect of dietary restriction (DR). This study examined the role of Foxo3 in lifespan extension and cancer suppression in DR mice. Wild-type (WT) and Foxo3-knockout heterozygous (^+/–^) and homozygous (^–/–^) mice were subjected to a 30% DR regimen initiated at 12 weeks of age. Control mice were fed *ad libitum* (AL) throughout the study. In contrast to WT mice, DR did not significantly extend the lifespan of Foxo3^+/–^ or Foxo3^–/–^ mice. However, DR reduced the prevalence of tumors at death in WT, Foxo3^+/–^, and Foxo3^–/–^ mice. These results indicate the necessity of Foxo3 for lifespan extension but not cancer suppression by DR. The findings in Foxo3^+/–^ mice contrast with those in Foxo1^+/–^ mice reported previously by our laboratory suggest differential regulation of cancer and lifespan by DR via Foxo1 and Foxo3.

Dietary restriction (DR) is known as a nongenetic intervention for the extension of lifespan and inhibition of diseases in aging animals (Weindruch & Walford, 1988). Although the molecular mechanisms by which DR extends lifespan have remained elusive for many years in mammals, recent studies using mutants of *Caenorhabditis elegans* have revealed genes required for the effect of DR (Greer & Brunet, 2009).

Daf-16, a forkhead transcription factor in *C. elegans*, was first identified as a mediator for lifespan extension induced by a reduction in insulin-like signaling (Daf-2 and Age-1 signaling; Kenyon *et al*., 1993). Subsequent studies also indicated the necessity of Daf-16 for the life-extending effect of DR in *C. elegans*, although the findings depend on the DR regimen (Greer & Brunet, 2009). Mammalian orthologs of Daf-16 include Foxo1, Foxo3, Foxo4, and Foxo6 (Greer & Brunet, 2005), which may be involved in the effects of DR. In fact, we have previously demonstrated that haploinsufficiency of Foxo1 diminishes the antineoplastic effect of DR in mice, indicating the necessity of Foxo1 for the effect of DR (Yamaza *et al*., 2010). However, compared with Wild-type (WT) mice, lifespan is extended by DR to the same extent in Foxo1^+/–^ mice. Our results indicate the involvement of Foxo1 in the antineoplastic effect of DR but not the regulation of lifespan. In *C. elegans*, four isoforms of Daf-16 have been isolated (Lin *et al*., 2001; Kwon *et al*., 2010). Among these isoforms, Daf-16a and Daf-16d/f are major regulators of lifespan under reduced insulin signaling, suggesting isoform-specific regulation of lifespan (Lin *et al*., 2001; Kwon *et al*., 2010). These findings prompted us to examine the role of Foxo3 in the effects of DR in mice.

In this study, male WT, Foxo3^+/–^, and Foxo3^–/–^ mice were subjected to 30% DR initiated at 12 weeks of age. The genetic background of the mice was C57BL6 (Miyamoto *et al*., 2007). Control mice were maintained under the *ad libitum* (AL) condition. Foxo3 mRNA expression levels were not affected by DR in various tissues of WT mice (Yamaza *et al*., 2010; Fig. S1). Compared with WT tissues, Foxo3 mRNA levels were reduced in Foxo3^+/–^ and Foxo3^–/–^ mouse tissues depending on the Foxo3 gene allele (Fig. S2). The Foxo3 protein abundance was also reduced by up to 50% in Foxo3^+/–^-DR mice compared with that in WT-DR mice (Fig. S3), whereas no alteration in the abundance of Foxo1 was found in Foxo3^+/–^ mice. The food intake by Foxo3^+/–^ and Foxo3^–/–^ mice was similar to those by WT mice under the AL condition, and thus, the daily allotments for each DR group were almost the same during the lifespan study (Fig. S4). The average body weights of WT, Foxo3^+/–^, and Foxo3^–/–^ mice were also similar under AL and DR conditions (Fig. S5).

Lifespans in WT- and Foxo3^+/–^-AL groups were equivalent, although the lifespan in Foxo3^–/–^-AL mice was slightly shorter compared with those of WT- and Foxo3^+/−^-AL groups (Fig.1; Fig. S6). DR extended lifespan in WT mice (*P* = 0.0011 by log-rank test; Fig.1A). However, there was no significant increase in the lifespans of Foxo3^+/−^ and Foxo3^−/−^ mice by DR (*P* = 0.8363 and *P* = 0.3150; Fig.1B,C). By comparing lifespans between DR groups, we found that WT-DR mice lived longer than Foxo3^+/−^- or Foxo3^−/−^-DR mice (*P* = 0.0060 and *P* = 0.0112; Fig.1D). Unlike WT mice, a Cox proportional hazards model also validated that DR did not extend lifespan in Foxo3^+/−^ mice (interaction between the genotype and diet effects (Genotype × Diet, *P* = 0.00269; Table S1).

**Fig 1:**
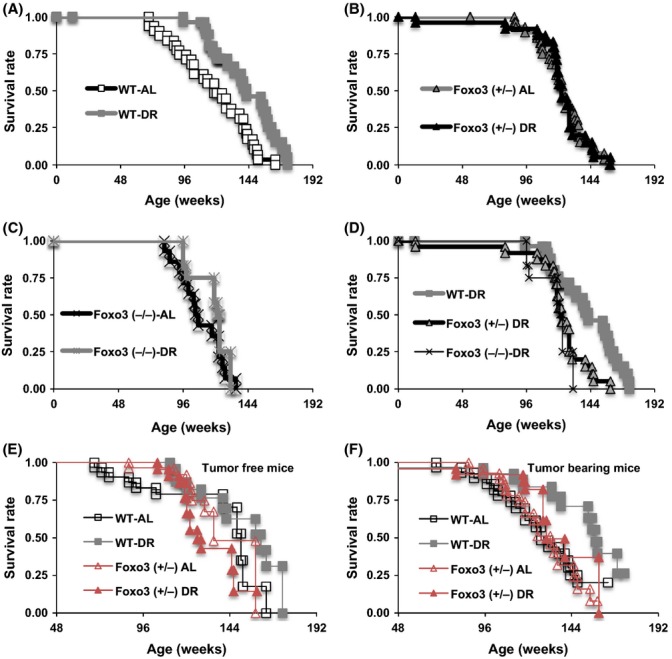
Survival curves of wild-type (WT), Foxo3^+/−^, and Foxo3^−/−^ male mice fed *ad libitum* (AL) or 30% dietary restriction (DR) diets. (A) DR significantly extends lifespan in WT mice (*P* = 0.0011 by the Log-rank test. (B, C). The effect of DR is abrogated in FoxO3^+/−^ and Foxo3^−/−^ mice (*P* = 0.8363 and *P* = 0.3150). (D) Comparison of lifespans between WT-DR, Foxo3^+/−^-DR, and Foxo3^−/−^-DR mice (*P* = 0.0060 in WT-DR vs. Foxo3^+/−^-DR mice; *P* = 0.0112 in WT-DR vs. Foxo3^−/−^-DR mice). The initial number of mice in each group was as follows; WT-AL, 31; WT-DR, 27; Foxo3^+/−^-AL, 29; Foxo3^+/−^-DR, 24; Foxo3^−/−^-AL, 14; Foxo3^−/−^-DR, 12). (E) Survival curves of tumor-free mice. DR appeared to affect the tumor-free lifespan of Foxo3^+/−^ mice in a different manner to WT mice (Genotype × Diet interaction, *P* = 0.0553 by the Cox proportional hazards model, Table S1). F) Survival curves of tumor-bearing mice. DR extended the lifespan of Foxo3^+/−^ and WT mice similarly (Diet, *P* = 0.0045; Genotype × Diet, *P* = 0.3131, Table S1).

Postmortem examination revealed that 66.7% of WT-AL mice had some type of tumor such as malignant lymphoma (Table1). In WT-DR mice, the tumor prevalence was reduced significantly compared with that in WT-AL mice (*P* = 0.024 by the likelihood ratio test). In Foxo3^+/−^ mice, DR also significantly decreased the tumor prevalence (*P* = 0.0263); the prevalence of tumor also appeared to be reduced by DR in Foxo3^−/−^ mice, although it was statistically insignificant probably because of the small numbers of mice examined.

**Table 1:** Prevalence of spontaneously occurring tumors at death in Foxo3^+/−^ and Foxo3^−/−^ mice

	WT-AL	WT-DR	Foxo3^+/−^-AL	Foxo3^+/−^-DR	Foxo3^−/−^-AL	Foxo3^−/−^-DR
Tumor+	20/30 (66.7%)	10/27 (37.0%)*	18/24 (75.0%)	10/23 (43.5%)*	10/15 (66.7%)	3/11 (27.3%)
ML	16	10	12	7	8	3
LC	2	1	7	1	2	0
HCC	3	0	5	1	1	0
Others	2	0	1	1	0	0

Tumor+, the number of mice with tumors/the number of mice examined.

* *P* < 0.05 vs. respective control AL groups by the likelihood ratio test. ML, malignant lymphoma. WT, Wild-type. DR, dietary restriction. LC, lung carcinoma or adenoma. HCC, hepatocellular carcinoma. Some mice had multiple tumors at death. Therefore, the sum of the numbers of mice with ML, LC, HCC, and the others exceeds the number of mice with tumor+.

Because DR reduced the prevalence of tumors in Foxo3^+/−^ mice, we also analyzed the lifespan data by tumor-free and tumor-bearing mice separately with the Cox proportional hazards model (Fig.1E,F; Table S1). Similar to all mice, among tumor-free mice, DR appeared to affect lifespan in Foxo3^+/−^ mice differently from that in WT mice (Genotype × Diet, *P* = 0.0553; Table S1). In contrast, DR reduced the mortality of tumor-bearing Foxo3^+/−^ and WT mice in a similar fashion (Diet, *P* = 0.0045; Genotype × Diet, *P* = 0.3131; Fig.1F and Table S1).

These findings suggest that Foxo3 is required for the life-extending effect of DR, but unnecessary for the antineoplastic effect of DR. The results in Foxo3^+/−^ mice contrast with those in Foxo1^+/−^ mice (Yamaza *et al*., 2010) suggest differential regulation of cancer and lifespan by DR via Foxo1 and Foxo3 (Fig. S7).

Paik *et al*. (2007) reported functional redundancy of Foxo1, Foxo3, and Foxo4 in the regulation of lifespan and cancer in mice under the AL condition. Single deletion of either Foxo1, Foxo3, or Foxo4 genes resulted in minor alterations in the incidence of neoplasms and lifespan; only triple knockout of Foxo1, Foxo3, and Foxo4 genes caused the cancer-prone phenotype and shortened lifespan. However, our study indicates the distinct roles of Foxo1 and Foxo3 genes under DR conditions. Natural roles of Foxo1 and Foxo3 might surface under harsh conditions such as DR, because each Foxo should be activated to adapt to these conditions and to protect cells from insults.

Finally, a group of single nucleotide polymorphisms in linkage disequilibrium within a coding region of Foxo3 (but not Foxo1) is reported to be associated with human longevity (Willcox *et al*., 2008). Therefore, our findings also suggest the presence of a common mechanism that regulates longevity and aging in a range of organisms. Dissection of the target genes of Foxo1 and Foxo3 under DR conditions might reveal precise pathways that regulate cancer and aging in mammals.

## Supporting Information

**Fig. S1.** Ratios of Foxo1, 3, and 4 mRNA expression levels in selected tissues between WT-DR and WT-AL mice.

**Fig. S2.** Expression levels of Foxo3 mRNA in selected tissues (A, Liver; B, Kidney; C, Peri-renal fat; D, Skeletal muscle).

**Fig. S3.** Expression levels of Foxo1 and Foxo3 proteins in the hippocampus and liver.

**Fig. S4.** Food intake in experimental groups of mice. The mean daily food consumption of Foxo3 (+/−) and Foxo3 (−/−) mice was similar to that of WT mice.

**Fig. S5.** Body weight of mice in the longevity study.

**Fig. S6**. Survival curves of wild-type (WT), Foxo3^+/−^, and Foxo3^−/−^ male mice fed ad libitum (AL).

**Fig. S7.** Hypothetical scheme of differential regulation of cancer and aging by DR via Foxo1 and 3.

**Table S1.** Effects of genotype and diet on lifespan: An analysis by a Cox proportional hazards model.

## References

[b1] Greer EL, Brunet A (2005). FOXO transcription factors at the interface between longevity and tumor suppression. Oncogene.

[b2] Greer EL, Brunet A (2009). Different dietary restriction regimens extend lifespan by both independent and overlapping genetic pathways in *C. elegans*. Aging Cell.

[b3] Kenyon C, Chang J, Gensch E, Rudner A, Tabtiang R (1993). A *C. elegans* mutant that lives twice as long as wild type. Nature.

[b4] Kwon E-S, Narasimhan SD, Yen K, Tissenbaum HA (2010). A new DAF-16 isoform regulates longevity. Nature.

[b5] Lin K, Hsin H, Libina N, Kenyon C (2001). Regulation of the *Caenorhabditis elegans* longevity protein DAF-16 by insulin/IGF-1 and germline signaling. Nat. Genet.

[b6] Miyamoto K, Araki KY, Naka K, Arai F, Takubo K, Yamazaki S, Matsuoka S, Miyamoto T, Ito K, Ohmura M, Chen C, Hosokawa K, Nakauchi H, Nakayama K, Nakayama KI, Harada M, Motoyama N, Suda T, HIrano A (2007). Foxo3a is essential for maintenance of the hematopoietic stem cell pool. Cell Stem Cell.

[b7] Paik J-H, Kollipara R, Chu G, Ji H, Xiao Y, Ding Z, Miao L, Tothova Z, Horner JW, Carrasco DR, Jiang S, Gilliland DG, Chin L, Wong WH, Castrillon DH, DePinho RA (2007). FoxOs are lineage-restricted redundant tumor suppressors and regulate endothelial cell homeostasis. Cell.

[b8] Weindruch R, Walford RL (1988). The Retardation of Aging and Disease by Dietary Restriction.

[b9] Willcox BJ, Donlon TA, He Q, Chen R, Grove JS, Yano K, Masaki KH, Willcox DC, Rodriguez B, Curb JD (2008). FOXO3A genotype is strongly associated with human longevity. Proc. Natl Acad. Sci. USA.

[b10] Yamaza H, Komatsu T, Wakita S, Kijogi C, Park S, Hayashi H, Chiba T, Mori R, Furuyama T, Mori N, Shimokawa I (2010). FoxO1 is involved in the antineoplastic effect of calorie restriction. Aging Cell.

