# Supplementary figures and images for: The life-extending effect of dietary restriction requires Foxo3 in mice
Source: Aging Cell. 2015 Mar 23;14(4):707–9. doi: 10.1111/acel.12340 (PMC4531086; doi:10.1111/acel.12340)

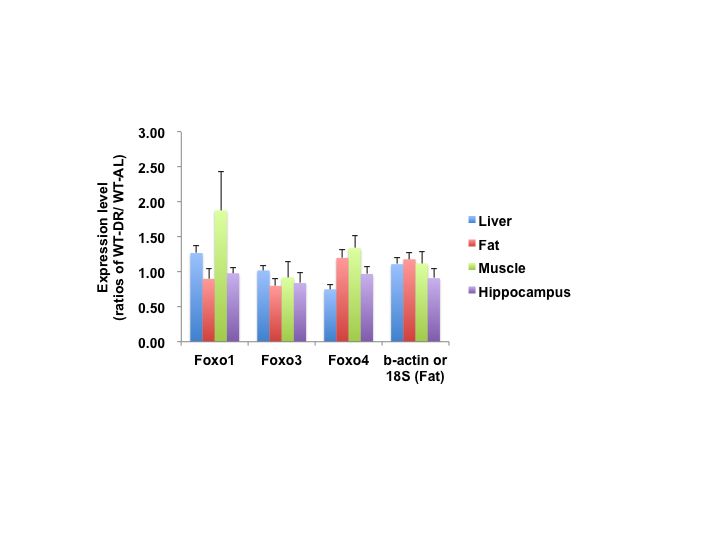

Supplement: Supplementary file 1 [file acel0014-0707-sd1.jpg]

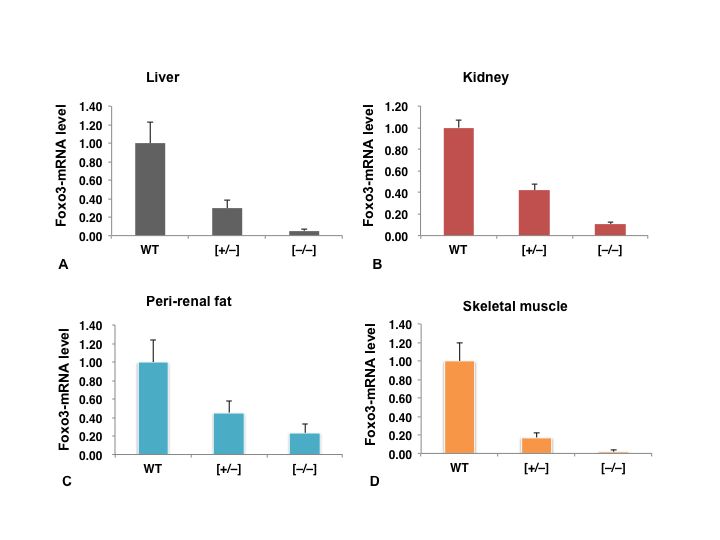

Supplement: Supplementary file 2 [file acel0014-0707-sd2.jpg]

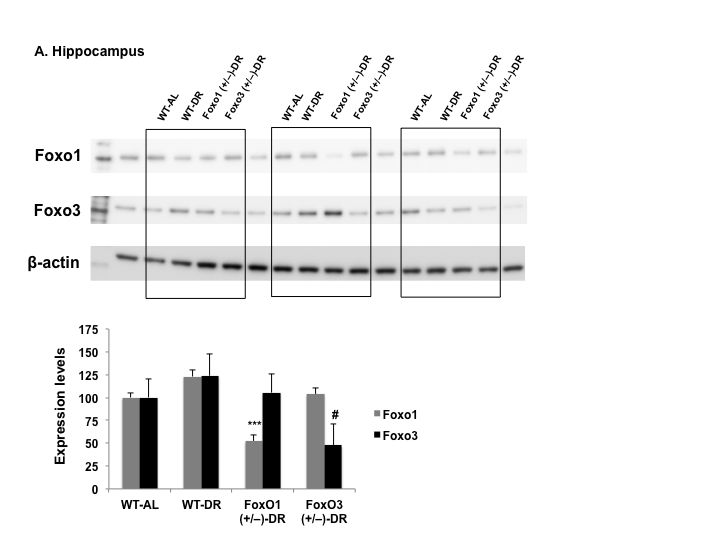

Supplement: Supplementary file 3 [file acel0014-0707-sd3.jpg]

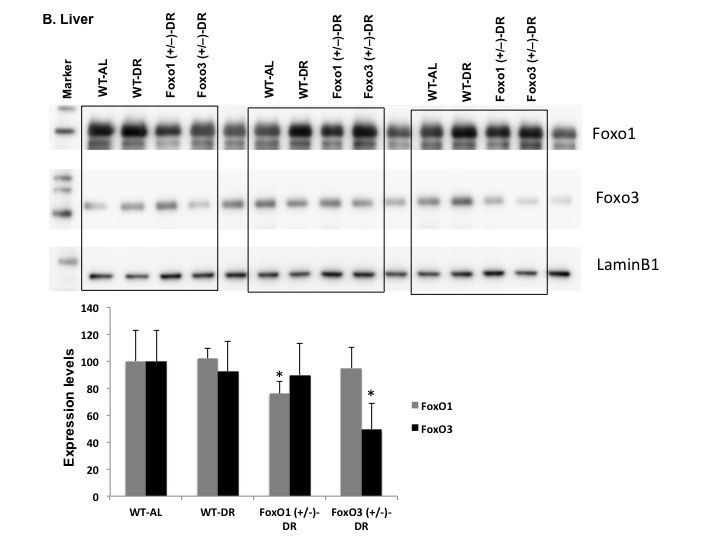

Supplement: Supplementary file 4 [file acel0014-0707-sd4.jpg]

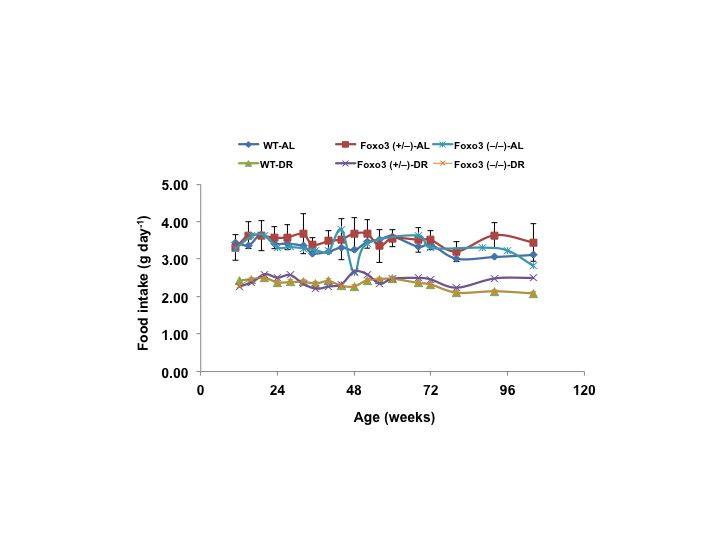

Supplement: Supplementary file 5 [file acel0014-0707-sd5.jpg]

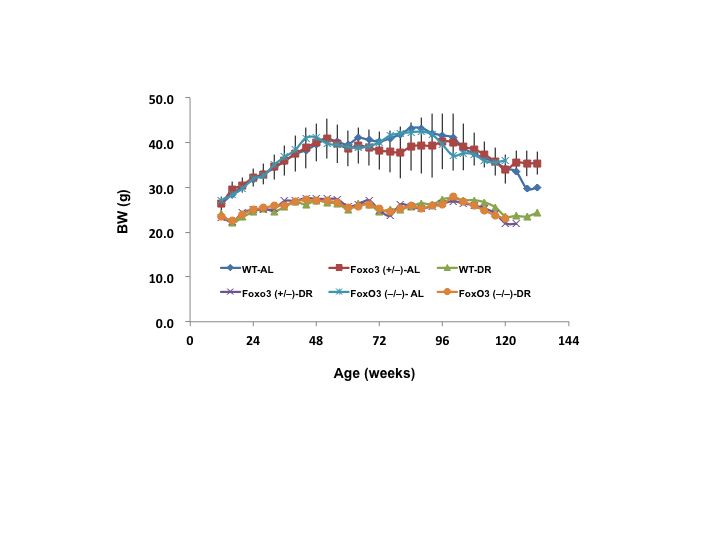

Supplement: Supplementary file 6 [file acel0014-0707-sd6.jpg]

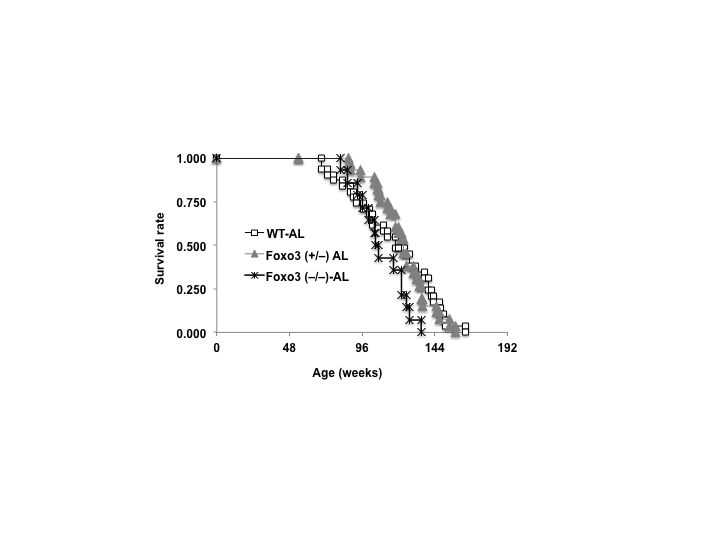

Supplement: Supplementary file 7 [file acel0014-0707-sd7.jpg]

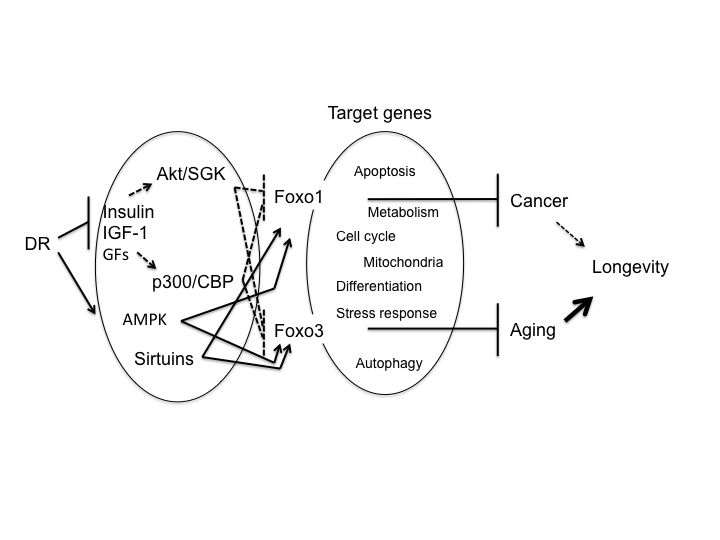

Supplement: Supplementary file 8 [file acel0014-0707-sd8.jpg]
