# Supplementary material for: The life-extending effect of dietary restriction requires Foxo3 in mice
Source: Aging Cell. 2015 Mar 23;14(4):707–9. doi: 10.1111/acel.12340 (PMC4531086; doi:10.1111/acel.12340)
Supplement: Supplementary file 9 [file acel0014-0707-sd9.docx]

Table S1. Effects of genotype and diet on lifespan: An analysis by a Cox proportional hazards model

|  |  | Risk ratio (95% CI) | p-value |
| --- | --- | --- | --- |
| All mice | Genotype | 1.64 (1.07–2.52) | 0.0234 |
|  | Diet | 0.59 (0.39–0.89) | 0.0118 |
|  | Genotype × Diet | – | 0.0269 |
|  |  |  |  |
| Tumor-free mice | Genotype | 1.76 (0.92–3.39) | 0.0863 |
|  | Diet | 0.96 (0.52–1.78) | 0.8916 |
|  | Genotype × Diet | – | 0.0553 |
|  |  |  |  |
| Tumor-bearing mice | Genotype | 1.40 (0.79–2.47) | 0.2521 |
|  | Diet | 0.45 (0.25–3.96) | 0.0045 |
|  | Genotype × Diet | – | 0.3131 |

Based on postmortem examination, we classified mice that died spontaneously as tumor-free or tumor-bearing mice. Genotype, Foxo3^+/–^ versus wild-type. Diet, DR versus AL. CI, confidence interval.
